# Beyond Slurry-Cast Supercapacitor Electrodes: PAN/MWNT Heteromat-Mediated Ultrahigh Capacitance Electrode Sheets

**DOI:** 10.1038/srep41708

**Published:** 2017-01-31

**Authors:** Jung Han Lee, Jeong A Kim, Ju-Myung Kim, Sun-Young Lee, Sun-Hwa Yeon, Sang-Young Lee

**Affiliations:** 1 Department of Energy Engineering, School of Energy and Chemical Engineering, Ulsan National Institute of Science and Technology (UNIST), Ulsan 689-798, Korea; 2 Department of Forest Products, Korea Forest Research Institute, Seoul 02455, Korea; 3 Energy Storage Lab., Korea Institute of Energy Research (KIER), Yuseong, Daejeon 305-343, Korea

## Abstract

Supercapacitors (SCs) have garnered considerable attention as an appealing power source for forthcoming smart energy era. An ultimate challenge facing the SCs is the acquisition of higher energy density without impairing their other electrochemical properties. Herein, we demonstrate a new class of polyacrylonitrile (PAN)/multi-walled carbon tube (MWNT) heteromat-mediated ultrahigh capacitance electrode sheets as an unusual electrode architecture strategy to address the aforementioned issue. Vanadium pentoxide (V_2_O_5_) is chosen as a model electrode material to explore the feasibility of the suggested concept. The heteromat V_2_O_5_ electrode sheets are produced through one-pot fabrication based on concurrent electrospraying (for V_2_O_5_ precursor/MWNT) and electrospinning (for PAN nanofiber) followed by calcination, leading to compact packing of V_2_O_5_ materials in intimate contact with MWNTs and PAN nanofibers. As a consequence, the heteromat V_2_O_5_ electrode sheets offer three-dimensionally bicontinuous electron (arising from MWNT networks)/ion (from spatially reticulated interstitial voids to be filled with liquid electrolytes) conduction pathways, thereby facilitating redox reaction kinetics of V_2_O_5_ materials. In addition, elimination of heavy metallic foil current collectors, in combination with the dense packing of V_2_O_5_ materials, significantly increases (electrode sheet-based) specific capacitances far beyond those accessible with conventional slurry-cast electrodes.

Ongoing surge in demand for electric vehicles (EVs), stationary energy storage systems (ESSs), and flexible portable electronics relentlessly pushes us to develop advanced rechargeable power sources affording reliable electrochemical performance and safety tolerance^1^,^2^. Among numerous power sources reported to date, supercapacitors (SCs) have garnered a great deal of attention as an appealing system to fulfill the aforementioned requirements owing to their exceptional rate capability, cycle life, electrochemical reversibility, and safety^3^,^4^,^5^. These advantageous characteristics of SCs are believed to be well-suited particularly for EV battery applications^6^,^7^,^8^. However, the relatively low energy density of SCs, compared to those of other power sources such as lithium-ion batteries, has posed a formidable challenge to their versatile applications. Of various SC systems reported to date, metal oxide-based SCs are featured with reversible Faradaic redox reactions, thus enabling significant increase in capacitance^9^,^10^,^11^. Previous studies on the metal oxide SCs have been mostly devoted to synthesis and engineering of electrode active materials themselves^12^,^13^,^14^,^15^, with a particular focus on improvement of their capacitance, electrical conductivity, and electrolyte accessibility.

Meanwhile, from the electrode architecture point of view, conventional electrodes are fabricated by slurry casting method, in which electrode active materials, carbon powder conductive additives, and polymer binders are simply piled up on top of metallic foil (or foam) current collectors^16^,^17^. Unfortunately, such a stereotypical electrode architecture often gives rise to nonuniform/sluggish transport of electrons and ions particularly in through-thickness direction of electrodes. Moreover, the inevitable use of heavy metallic foil current collectors has made it difficult for us to further increase energy density in a fixed electrode volume/weight. One promising way to resolve these issues is the removal of heavy metallic current collectors in the electrodes. A number of studies have been reported for the development of metallic foil current collector-free electrodes for SCs^11^,^18^,^19^ and also lithium-ion batteries^20^,^21^,^22^,^23^. However, most of these previous works have combined electrode materials with pre-formed three-dimensional (3D) porous scaffolds, thus resulting in low active-mass loading per unit electrode area.

Here, we present a new class of metallic foil current collector-free, polyacrylonitrile (PAN)/multi-walled carbon tube (MWNT) heteromat-mediated ultrahigh capacitance electrode sheets as an unusual electrode architecture strategy to address the aforementioned long-standing challenge of SC electrodes. As a proof-of-concept for this approach, vanadium pentoxide (V_2_O_5_) is chosen as a model electrode material. V_2_O_5_ has been investigated due to its natural abundance, low cost, and various oxidation states (V^2+^ to V^5+^) suitable for realization of higher pseudo-capacitance^24^,^25^,^26^. However, V_2_O_5_ suffers from low electronic conductivity (10^−2^ to 10^−3^ S cm^−1^), which remains a critical challenge to its application to SC electrodes.

The heteromat V_2_O_5_ electrode sheets presented herein are composed of densely-packed V_2_O_5_ materials in intimate contact with the MWNTs and the PAN nanofibers. The MWNTs offer highly-interconnected electronic networks and also serve as an alternative current collector. The PAN nanofibers act as a mechanically-reinforcing skeleton and also an one-dimensional (1D)-shaped electrode binder. Notably, the V_2_O_5_ electrode sheets are produced through one-pot fabrication based on concurrent electrospraying (for V_2_O_5_ precursor (=VOC_2_O_4_)/MWNT) and electrospinning (for PAN nanofiber) followed by calcination. Benefiting from the aforementioned material/architecture uniqueness, the heteromat V_2_O_5_ electrode sheets offer 3D bicontinuous electron/ion conduction pathways, thereby facilitating redox reaction kinetics of V_2_O_5_ materials. Moreover, the removal of heavy metallic foil current collectors, in association with compact packing of V_2_O_5_ materials, enables a remarkable increase in (electrode sheet-based) specific capacitances, which lie far beyond those achievable with conventional slurry-cast electrodes.

## Results

### One-pot fabrication and structural/physicochemical characterization of heteromat V_2_O_5_ electrode sheets

**Figure 1:**
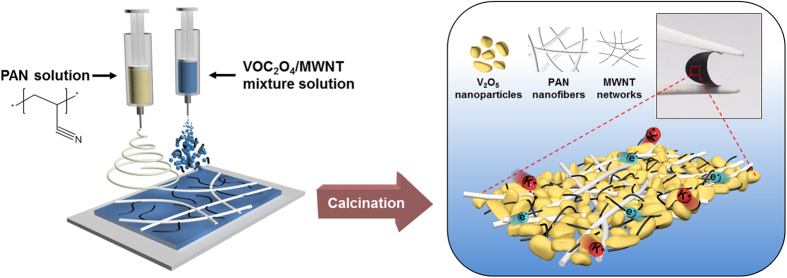
Schematic illustration showing the fabrication procedure and morphological uniqueness of heteromat V_2_O_5_ electrode sheets. The one-pot fabrication based on concurrent electrospraying (for V_2_O_5_ precursor/MWNT) and electrospinning (for PAN nanofiber) followed by calcination was depicted.

The self-standing, metallic foil current collector-free heteromat V_2_O_5_ electrode sheets were produced through the concurrent electrospraying (for V_2_O_5_ precursor (=VOC_2_O_4_)/MWNT) and electrospinning (for PAN nanofiber) followed by calcination in air. This one-pot fabrication procedure of the V_2_O_5_ electrode sheet, along with its morphological uniqueness, was schematically illustrated in Fig. 1.

**Figure 2:**
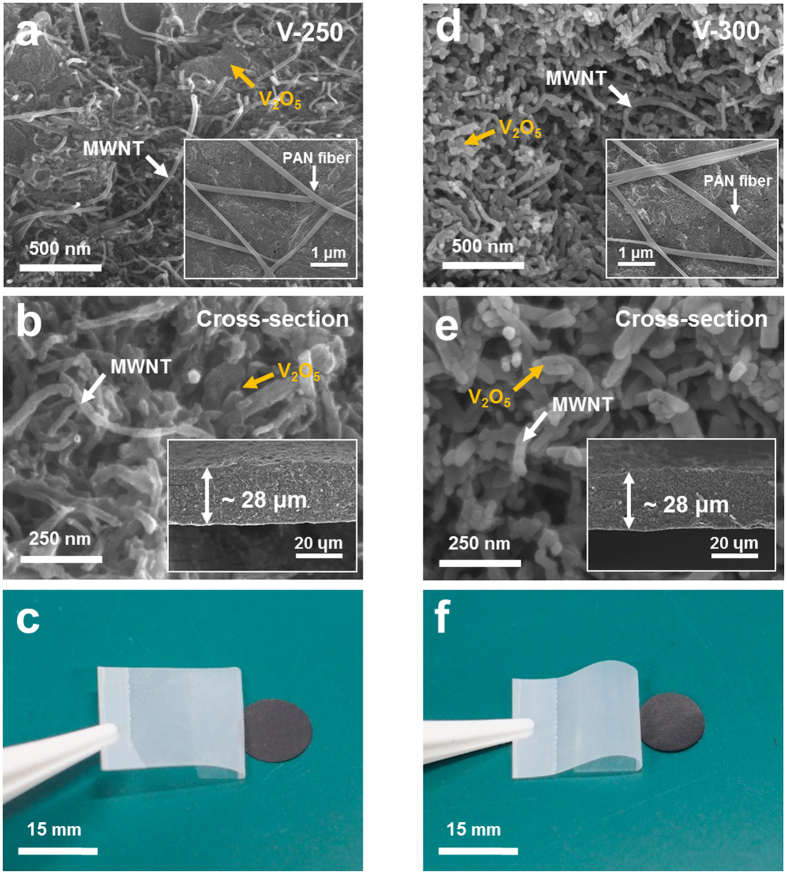
Morphological characterization of: (**a**–**c**) V-250 and (**d**–**f**) V-300 electrode sheets. (**a**,**d**) SEM images (surface) showing the good dispersion state of V_2_O_5_, MWNTs and PAN nanofibers, wherein insets are low-magnification view. (**b**,**e**) SEM images (cross-section). (**c**,**f**) A tape test using commercial 3 M scotch^®^ tape.

The structure and physicochemical properties of the V_2_O_5_ electrode sheets were investigated as a function of calcination temperature (250 and 300 °C) that is expected to affect formation of amorphous or crystalline structure of the resulting V_2_O_5_ materials. The SEM images (Fig. 2a (surface) and b (cross-section)) of the V_2_O_5_ electrode sheet calcined at 250 °C (denoted as “V-250 electrode sheet”) showed that the V_2_O_5_, MWNTs, PAN nanofibers are well mingled together and the thickness of the electrode sheet was approximately 28 μm. The V_2_O_5_ clusters were densely packed and in close contact with the electrosprayed MWNTs under the presence of PAN nanofibers. Meanwhile, the morphology of the V_2_O_5_ electrode sheet (“V-300 electrode sheet”, thickness ~28 μm) calcined at 300 °C was characterized (Fig. 2d and e). A notable structural feature of the V-300 electrode sheet is the finely-dispersed V_2_O_5_ nanoparticles. This structural uniqueness of the V-250 and V-300 electrode sheets was further verified by analyzing TEM images (Supplementary Fig. S1). The MWNTs, together with the V_2_O_5_, were uniformly dispersed without serious aggregation, resulting in the highly interconnected electronic networks. In addition to the well-developed MWNT electronic networks, the spatially reticulated interstitial voids, which will be filled with electrolyte and thus act as ion-conducting pathways, were formed in the V-250 and V-300 electrode sheets. The difference in the V_2_O_5_ morphology between the two electrode sheets will be discussed in the following section, together with in-depth structural characterization.

Both the heteromat V_2_O_5_ electrode sheets exhibited the compact packing of V_2_O_5_ materials, which is anticipated to enable the realization of (electrode sheet-based) high specific capacitance. From the TGA result (Supplementary Fig. S2) and the selective etching of PAN (dimethylformamide (DMF) was used as an etching agent), the composition ratio of the V-250 and V-300 electrode sheets was estimated to be (V_2_O_5_/MWNT)/PAN = (48/33)/19 (w/w/w). The effect of the composition ratio of the electrode sheets on their strucutre and electrochemical performance was examined. Below the MWNT content of 30 wt%, the resulting electrode sheets did not show significant improvement in the cell performance, which appeared similar to a control sample (fabricated through a conventional slurry cast method). Meanwhile, when the MWNT content was larger than 33 wt%, we failed to prepare V_2_O_5_/MWNT suspensions because electrospraying nozzles were frequently clogged. The structural robustness of the V-250 and V-300 electrode sheets was examined using peel-off test with 3 M scotch^®^ tape. Neither detachment nor disintegration of the electrode components was observed for both electrodes (Fig. 2c and f), demonstrating that the V_2_O_5_ materials, MWNTs, and PAN nanofibers were tightly held together even in the absence of conventional polymer binders and metallic foil current collectors.

**Figure 3:**
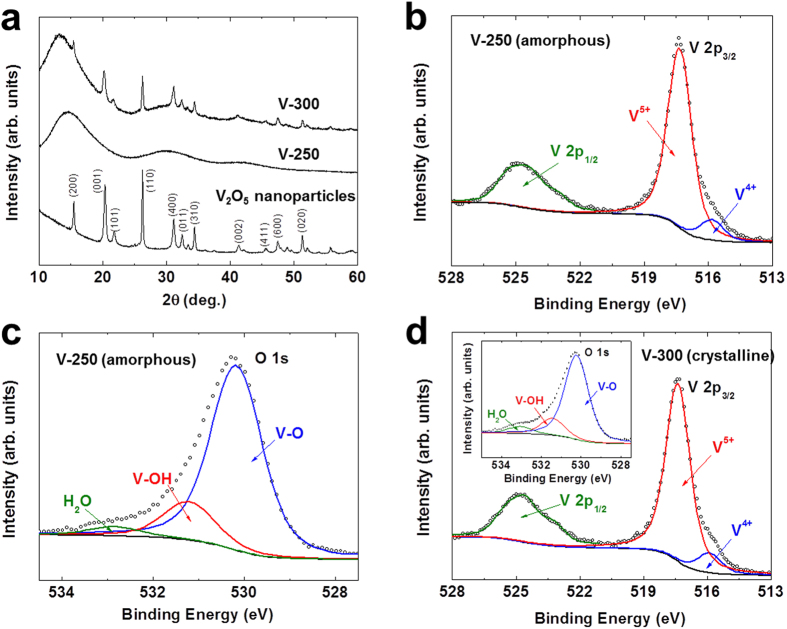
Structural (amorphous vs. crystalline) analysis of V-250 and V-300 electrode sheets. (**a**) XRD patterns showing the characteristic peaks ascribed to orthorhombic V_2_O_5_ with layered shcherbinaite structure (*Pmn*2_1_). (**b**) XPS spectra showing the characteristic V 2*p*_1/2_ (525 eV) and V 2*p*_3/2_ (517 eV) peaks of V-250 electrode sheet. (**c**) XPS O 1 *s* peaks assigned to V-O (530 eV) and V-OH (531 eV) of V-250 electrode sheet. (**d**) XPS V 2*p*_1/2_ and V 2*p*_3/2_ peaks (an inset is O 1 *s* peaks) of V-300 electrode sheet.

The one-pot synthesized V_2_O_5_ active materials in the V-250 and V-300 electrode sheets were characterized in more detail. Figure 3a shows XRD patterns of the V-250 and V-300 electrode sheets, along with those of control V_2_O_5_ nanoparticles (which were synthesized using the same VOC_2_O_4_ precursor and calcination condition employed for the V-300 electrode sheet). The control V_2_O_5_ nanoparticles showed the characteristic XRD peaks assigned to orthorhombic V_2_O_5_ with layered shcherbinaite structure (*Pmn*2_1_)^27^,^28^. It is of note that the XRD peaks of the V_2_O_5_ in the V-300 electrode sheet appear well-matched with those of the control V_2_O_5_, verifying the synthesis of crystalline V_2_O_5_ materials in the V-300 electrode sheet. In comparison, no appreciable XRD peaks were observed at the V-250 electrode sheet, revealing the formation of amorphous V_2_O_5_ materials.

The V_2_O_5_ materials in the V-250 and V-300 electrode sheets were further elucidated using XPS spectra. The characteristic V 2*p*_1/2_ (525 eV) and V 2*p*_3/2_ (517 eV) peaks^23^,^29^ were observed at the V-250 electrode sheet (Fig. 3b). Intriguingly, the V 2*p*_3/2_ peaks were resolved into two contributions, V^4+^ and V^5+^. The relatively higher intensity of the V^5+^ peaks indicates that a majority of the vanadium are V^5+^ state (i.e., corresponding to V_2_O_5_). In addition, the XPS O 1 *s* peaks^30^ assigned to V-O (530 eV) and V-OH (531 eV) were detected at the V-250 electrode sheet (Fig. 3c). The aforementioned XPS spectra were also observed at the V-300 electrode sheet, demonstrating the presence of V_2_O_5_ materials (Fig. 3d). This structural characterization exhibits that the amorphous and crystalline V_2_O_5_ materials were successfully one-pot synthesized in the V-250 and V-300 electrode sheets, respectively.

An essential prerequisite to enable facile electrochemical reaction in rechargeable power sources is construction of well-developed electronic/ionic pathways. The electronic conductivity and electrolyte accessibility of the V-250 and V-300 electrode sheets were compared with those of a control V_2_O_5_ electrode sheet that was fabricated using a conventional slurry casting method (V_2_O_5_/carbon black additive/PVdF binder = 70/20/10 (w/w/w) on a Ni foil current collector, Supplementary Fig. S3). The V-250 electrode sheet presented the higher electronic conductivity (=4.4 S cm^−1^, Supplementary Fig. S4a) than the control V_2_O_5_ electrode sheet (=1.3 S cm^−1^). This facile electron conduction is attributed to the highly-interconnected MWNT electronic networks and the removal of conventional polymeric binders (that may partially shield V_2_O_5_ materials and conductive pathways). To check influence of the PAN nanofibers calcined at 300 °C on electronic conductivity, one control sheet solely comprising PAN nanofibers was fabricated. The PAN nanofiber sheet showed no detectable level of electronic conductivity (~0 S cm^−1^) after the calcination at 300 °C in air, revealing that the PAN nanofibers themselves in the V-250 and V-300 electrode sheets would remain electronically inert.

The V-250 and V-300 electrode sheets showed the higher porosity (~29%) than the control V_2_O_5_ electrode sheet (~23%). Furthermore, the spatially reticulated interstitial void channels, in combination with the polar PAN nanofibers, facilitated capillary intrusion of liquid electrolyte (=2 M KCl aqueous electrolyte) into the V-250 and V-300 electrode sheets (Supplementary Fig. S4b), indicating the better electrolyte accessibility. These results demonstrate that the PAN/MWNT heteronanomat-mediated architecture of the V-250 and V-300 electrode sheets allowed for the construction of highly interconnected dual (i.e., electron/ion) conduction pathways, which is thus expected to boost up Faradaic redox reaction kinetics.

### Electrochemical characterization of heteromat V_2_O_5_ (V-250 and V-300) electrode sheets

**Figure 4:**
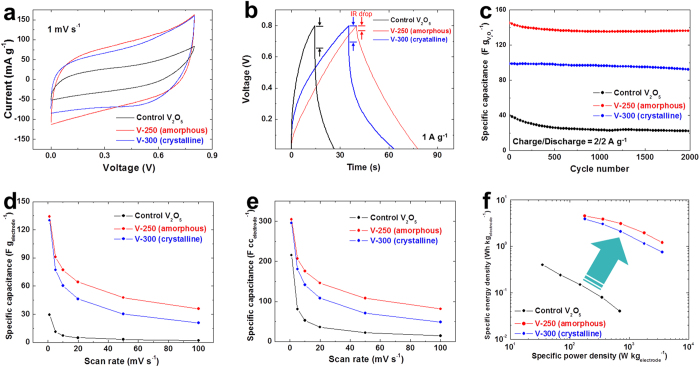
Electrochemical characterization of V-250, V-300, and control V_2_O_5_ electrode sheets. (**a**) Cyclic voltammetry (CV) curves (scan rate = 1 mV s^−1^) showing typical Faradaic pseudocapacitive behavior. (**b**) Galvanostatic charge/discharge (GCD) profiles (current density = 1.0 A cm^−2^). (**c**) Comparison in the cycling performance (up to 2,000 cycles) between the different electrode sheets at a current density of 2.0 A g^−1^. (**d**) Comparison in the (electrode sheet-based) specific gravimetric capacitance (F g_electrode_^−1^) between the different electrode sheets as a function of scan rate (1–100 mV s^−1^). (**e**) Comparison in the (electrode sheet-based) specific volumetric capacitance (F cc_electrode_^−1^) between the different electrode sheets as a function of scan rate (1–100 mV s^−1^). (**f**) Ragone plots (specific gravimetric energy density (=Wh kg_electrode_^−1^) vs. specific gravimetric power density (=W kg_electrode_^−1^)) of SC cells, wherein the cell weight was determined by solely considering the electrode sheet weight.

The electrochemical performance of the V-250/V-300 electrode sheets was investigated with pouch-type symmetric cells (incorporating 2 M KCl aqueous electrolyte). The electrochemical reaction in V_2_O_5_ electrodes is can be expressed as follows^31^:


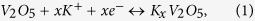


wherein *x* is the mole fraction of reacted K^+^ ions. The cyclic voltammetry (CV) curves (measured at a scan rate of 1 mV s^−1^) showed typical Faradaic pseudocapacitive behavior (Fig. 4a). The V-250 electrode sheet presented the higher (V_2_O_5_ powder weight-based) specific gravimetric capacitance (=266 F g_V2O5_^−1^) than the V-300 (=259 F g _V2O5_^−1^) and the control V_2_O_5_ electrode sheet (=240 F g_V2O5_^−1^). This higher capacitance of the V-250 electrode sheet became more apparent with increasing scan rate (Supplementary Figs S5 and S6) To exactly estimate the specific gravimetric capacitance of V_2_O_5_ active materials, contribution of MWNTs themselves (Supplementary Fig. S7) was excluded.

The capacitance of SCs is known to depend on measurement condition (specifically, symmetric two-electrode vs. three-electrode configuration). Theoretically, the capacitance estimated from the three-electrode configuration is four times higher than that from the symmetric two-electrode system^4^. In addition, the three-electrode configuration tends to overestimate specific capacitances, while the two-electrode analysis shows opposite behavior^24^. It is of note that under the symmetric two-electrode configuration, the V-250 electrode sheet showed the higher specific gravimetric capacitance than previously reported V_2_O_5_/CNT composite electrodes (Supplementary Table S1)^32^,^33^,^34^,^35^. This excellence in the capacitance of the V-250 electrode sheet was further verified by conducting galvanostatic charge-discharge (GCD) tests. The symmetric triangular-shaped charge/discharge profiles were found at all the electrode sheets over a wide range of current densities (0.5–5.0 A g^−1^) (Supplementary Fig. S8). The comparison in the GCD profiles (measured at 1.0 A g^−1^) between the different electrode sheets (Fig. 4b) exhibited that the V-250 electrode sheet presented the smaller IR drop (=0.03 V) and longer charge/discharge time (=40/39 s) as compared to the control V_2_O_5_ electrode sheet (=0.15 V and 14/12 s) and the V-300 electrode sheet (=0.1 V and 34/30 s). Moreover, the higher capacitance of the V-250 electrode sheet was maintained over 2,000 charge/discharge cycles at a current density of 2.0 A g^−1^ (Fig. 4c), manifesting the long-term cycling stability. The superior electrochemical performance of the V-250/V-300 electrode sheets over the control V_2_O_5_ electrode sheet is due to the PAN/MWNT heteronanomat-mediated structural uniqueness that allows 3D-bicontinuous electron/ion conduction. Meanwhile, amorphous V_2_O_5_ materials are known to provide higher capacitance than crystalline counterparts because their redox reaction occurs not only on the surface but also inside the bulk^5^,^26^,^36^. The aforementioned comparison in the specific gravimetric capacitance between the V-250 and V-300 electrode sheets appeared well consistent with the previously reported results.

It is again underlined that the V-250 electrode sheet does not contain heavy nickel (Ni) foil current collectors, which is thus anticipated to beneficially affect (electrode sheet-based) specific gravimetric/volumetric capacitance. The V-250 one showed the substantial reduction in the total areal weight, as compared to the control V_2_O_5_ electrode sheet (Supplementary Fig. S9). The areal active-mass loading (that exclusively considers the weight of V_2_O_5_ materials) of the V-250 electrode sheet (=2.0 mg cm^−2^) appeared negligibly different from that of the control V_2_O_5_ one (=2.3 mg cm^−2^), revealing that the lower weight of the V-250 electrode sheet is mainly due to the removal of the heavy Ni foil current collector (=23 mg cm^−2^). As a consequence, the V-250 electrode sheet exhibited the significant improvement in the specific gravimetric capacitance expressed as capacitance per electrode sheet weight (=F g_electrode_^−1^) (e.g., 134 F g_electrode_^−1^ at a scan rate of 1 mV s^−1^) than the conventional V_2_O_5_ electrode sheet (=29 F g_electrode_^−1^) over a wide range of scan rates (Fig. 4d). In addition, the increase in the specific volumetric capacitance expressed as capacitance per electrode sheet volume (=F cc_electrode_^−1^) was shown in Fig. 4e. The aforementioned superior electrochemical performance (shown in Fig. 4a–e) of the V-250 electrode sheet compared to the conventional V_2_O_5_ electrode sheet was further confirmed by the lower cell impedance (Supplementary Fig. S10), underscoring the advantageous effect of the PAN/MWNT heteromat electrode architecture on the cell performance. Notably, the V-250 and V-300 electrode sheets showed the significantly lower bulk resistance (at the highest frequency region) than the control V_2_O_5_ electrode sheet, demonstrating the more facilitated ion transport owing to their well-developed 3D continuous ion conduction channels.

The above-mentioned substantial improvement in the redox reaction kinetics (enabled by the PAN/MWNT heteronanomat-mediated 3D bicontinuous electron/ion conduction pathways) and the specific gravimetric/volumetric capacitance (enabled by the removal of heavy Ni foil current collectors and also dense packing of V_2_O_5_ materials) of the V-250 electrode sheet was further highlighted by analyzing the Ragone plot, in which the cell weight was determined by solely considering the electrode sheet weight. Figure 4f verified that the V-250 electrode sheet exhibited the remarkable increase in the (electrode sheet-based) specific gravimetric energy (=Wh kg_electrode_^−1^)/power (=W kg_electrode_^−1^) densities far beyond those accessible with the control V_2_O_5_ electrode sheet (fabricated by a conventional slurry casting method), underscoring its potential benefits as an exceptional high-energy/high-power density power source.

## Discussion

In summary, we presented the metallic foil current collector-free, PAN/MWNT heteromat-mediated V_2_O_5_ ultrahigh capacitance electrode sheets for use in SCs. The heteromat V_2_O_5_ electrode sheets were produced through the concurrent electrospraying (for V_2_O_5_ precursor/MWNT) and electrospinning (for PAN nanofiber) followed by the calcination. Notably, this one-pot fabrication of the V_2_O_5_ electrode sheet (starting directly from V_2_O_5_ precursor) allowed for dense packing of the resulting V_2_O_5_ materials in close contact with the MWNTs and PAN nanofibers. The heteromat V_2_O_5_ electrode sheet calcined at 250 °C yielded the amorphous V_2_O_5_, thus providing the higher capacitance than the heteromat V_2_O_5_ electrode one calcined at 300 °C (containing the crystalline V_2_O_5_). Driven by the material/architecture uniqueness, the heteromat V_2_O_5_ electrode sheets offered the 3D bicontinuous electron/ion conduction pathways, eventually facilitating the redox reaction kinetics of V_2_O_5_ materials. Furthermore, the removal of heavy Ni foil current collectors, in association with the compact packing of V_2_O_5_ materials, substantially increased the (electrode sheet-based) specific gravimetric electrode capacitances (e.g., 134 F g_electrode_^−1^ for the V-250 electrode sheet vs. 29 F g_electrode_^−1^ for the control V_2_O_5_ electrode sheet, at a scan rate of 1 mV s^−1^). As a consequence, the V_2_O_5_ electrode sheet enabled the shift of the specific energy/power densities to higher values in the Ragone plot, which lie far beyond those achievable with conventional slurry casting-based electrode technologies. The heteromat V_2_O_5_ electrode sheets based on the concept of “one-pot fabrication of electrode sheets directly from electrode material precursors” can be suggested as a facile and versatile platform technology (readily applicable to other electrode materials) and opens a new route towards high-energy/high-performance SC electrodes.

## Methods

### One-pot fabrication of PAN/MWNT heteromat-mediated V_2_O_5_ electrode sheets (directly from V_2_O_5_ precursors)

The V_2_O_5_ electrode sheets were produced through one-pot fabrication based on the concurrent electrospraying (for VOC_2_O_4_ (=V_2_O_5_ precursor)/MWNT) and electrospinning (for PAN nanofiber) followed by calcination in air. Firstly, in order to prepare the VOC_2_O_4_/MWNT mixture solution, 0.5 g of V_2_O_5_ powders (Aldrich) and 1.2 g of oxalic acid (Aldrich) were dissolved in 20 mL distilled water at room temperature for 3 h, yielding the VOC_2_O_4_ solution. Subsequently, 0.25 g of MWNT (CNT150, Hanwha), 0.12 g of PVP (molecular weight = 55,000 g mol^−1^, Aldrich), 20 mL of ethanol were added into the VOC_2_O_4_ solution under sonication. PAN (molecular weight = 150,000 g mol^−1^, Aldrich) was dissolved in dimethylformamide (DMF) at 80 °C for 12 h to prepare 10 wt.% PAN solution. The VOC_2_O_4_/MWNT and the PAN solutions were subjected to concurrent electropraying/electrospinning through different nozzles at room temperature. The working voltages/ejection rates were 10/10 (kV/μL min^−1^) for the electrospinning and 20/120 (kV/μL min^−1^) for the electrospraying, respectively. The resulting mixture mat was dried at 80 °C for 24 h and then heat-treated in air at 250 or 300 °C for 5 h to allow calcination of V_2_O_5_ directly inside the mixture mat, leading to a self-standing V_2_O_5_ electrode sheet. To prepare a control electrode sheet, bulk V_2_O_5_ nanoparticles were synthesized using the same VOC_2_O_4_ solution and the calcination process (300 °C for 5 h). The synthesized V_2_O_5_ nanoparticles were mixed with carbon black additives and polyvinylidene fluoride (PVdF) binder on a Ni foil current collector. The composition ratio of the control electrode sheet was V_2_O_5_/carbon black additive/PVdF binder = 70/20/10 (w/w/w).

### Structural characterization of heteromat V_2_O_5_ electrode sheets

The morphology of the heteromat V_2_O_5_ electrode sheet was investigated using field emission scanning electron microscopy (FE-SEM) (S-4800, Hitachi). The amorphous/crystalline phases of V_2_O_5_ materials in the cathode sheet were analyzed by X-ray diffraction (XRD) (D/MAZX 2500 V/PC) measurement using Cu Kα radiation. The composition ratio of the V_2_O_5_ electrode sheet was determined from thermogravimetric analysis (TGA) measurement (SDT Q600, TA Instruments) at a heating rate of 10 °C min^−1^ under air atmosphere. The electronic conductivity of the V_2_O_5_ electrode sheet was examined using 4-point probe point technique (CMT-SR1000N, Advanced Instrument Technology). The surface structure of the V_2_O_5_ electrode sheet was elucidated by X-ray photoelectron spectroscopy (XPS) (ThermoFisher) with focused monochromatized Al Kα radiation. The porosity of the V_2_O_5_ electrode sheets was estimated by measuring its density difference before and after solvent (n-butanol) uptake^37^.

### Electrochemical performance of heteromat V_2_O_5_ electrode sheets

The electrochemical performance of the heteromat V_2_O_5_ electrode sheet was characterized using a pouch-type symmetric cell, in which the V_2_O_5_ electrode sheet was assembled with a polypropylene (PP) separator (Celgard 3501) and 2 M KCl aqueous electrolyte. The cyclic voltammetry (CV), galvanostatic charge-discharge (GCD) and electrochemical impedance spectroscopy (EIS) measurements of the V_2_O_5_ electrode sheet were performed with a potentiostat/galvanostat (VSP classic, Bio-Logic).

## Additional Information

**How to cite this article**: Lee, J. H. *et al*. Beyond Slurry-Cast Supercapacitor Electrodes: PAN/MWNT Heteromat-Mediated Ultrahigh Capacitance Electrode Sheets. *Sci. Rep.* **7**, 41708; doi: 10.1038/srep41708 (2017).

**Publisher's note:** Springer Nature remains neutral with regard to jurisdictional claims in published maps and institutional affiliations.

## Supplementary Material

Supplementary Information
