# Supplementary material for: Beyond Slurry-Cast Supercapacitor Electrodes: PAN/MWNT Heteromat-Mediated Ultrahigh Capacitance Electrode Sheets
Source: Sci Rep. 2017 Jan 31;7:41708. doi: 10.1038/srep41708 (PMC5282478; doi:10.1038/srep41708)
Supplement: Supplementary Information [file srep41708-s1.doc]

**Supplementary Information**

**Beyond Slurry-Cast Supercapacitor Electrodes: PAN/MWNT Heteromat-Mediated Ultrahigh Capacitance Electrode Sheets**

Jung Han Lee^a†^, Jeong A Kim^a†^, Ju-Myung Kim^a^, Sun-Young Lee^b^, Sun-Hwa Yeon^c^,* and Sang-Young Lee^a^,*

^a^*Department of Energy Engineering, School of Energy and Chemical Engineering,*

*Ulsan National Institute of Science and Technology (UNIST), Ulsan 689-798, Korea*

^b^*Department of Forest Products, Korea Forest Research Institute, Seoul 02455, Korea*

^c^*Energy Storage Lab., Korea Institute of Energy Research (KIER),*

*Yuseong, Daejeon 305-343, Korea*

**CORRESPONDING AUTHOR FOOTNOTE:**

* Dr. Sun-Hwa Yeon

E-mail: ys93@kier.re.kr, Telephone +82-42-860-3763, Fax +82-42-860-3747

* Prof. Sang-Young Lee

E-mail: syleek@unist.ac.kr, Telephone +82-52-217-2948, Fax +82-52-217-2019

^†^ These authors contributed equally.

**Figure S1**. TEM images of the V-250 and V-300 electrode sheets. The well-established MWNT electronic networks and spatially reticulated interstitial voids were observed.

**Figure S2.** TGA profiles of V-250 and V-300 electrode sheets. From these TGA results and the selective etching of PAN (dimethylformamide (DMF) was used as an etching agent), the composition ratio of the electrode sheets was estimated to be (V_2_O_5_/MWNT)/PAN = (48/33)/19 (w/w/w).

**Figure S3.** SEM images of control V_2_O_5_ electrode sheet (V_2_O_5_ nanoparticles/carbon black additive/PVdF binder = 70/20/10 (w/w/w) on a Ni foil current collector, fabricated by conventional slurry casting method): (a) Surface. (b) Cross-section. (c) Cross-section (high-magnification).

**Figure S4.** Comparison in the physical properties between the V-250 and control V_2_O_5_ electrode sheet: (a) Electronic conductivity. (b) Electrolyte (= 2 M KCl aqueous electrolyte) wettability (expressed as electrolyte-immersion height).

**Figure S5.** Comparison in the (V_2_O_5_ powder weight-based) specific gravimetric capacitance (F g_V2O5_^-1^) between the electrode sheets as a function of scan rate (1 – 100 mV s^−1^).

**Figure S6.** Cyclic voltammetry (CV) curves of the electrode sheets over a wide range of scan rates (1 – 100 mV s^−1^) of: (a) V-250; (b) V-300; (c) control V_2_O_5_.

**Figure S7.** Specific gravimetric capacitance of the MWNT electrode sheet (MWNTs/PAN nanofibers = 61/29 (w/w), without V_2_O_5_ powders) over a wide range of scan rates (1 – 100 mV s^−1^).

**Figure S8.** Galvanostatic charge/discharge (GCD) profiles of the electrode sheets over a wide range of current densities (0.5 – 5.0 A g^−1^) of: (a) V-250; (b) V-300; (c) control V_2_O_5_.

**Figure S9.** Comparison in the areal mass (mg cm_electrode_^-2^) between the V-250 and control V_2_O_5_ electrode sheets in terms of their components.

**Figure S10**. AC impedance spectra of the V-250, V-300, and control V_2_O_5_ sheets.

**Table S1.** Comparison in the specific capacitance between this work and the previously reported V_2_O_5_/CNT composites.


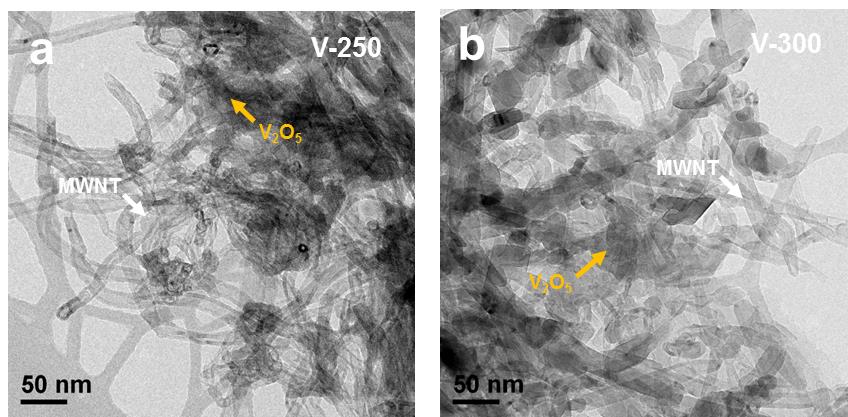


**Figure S1**. TEM images of the V-250 and V-300 electrode sheets. The well-established MWNT electronic networks and spatially reticulated interstitial voids were observed.


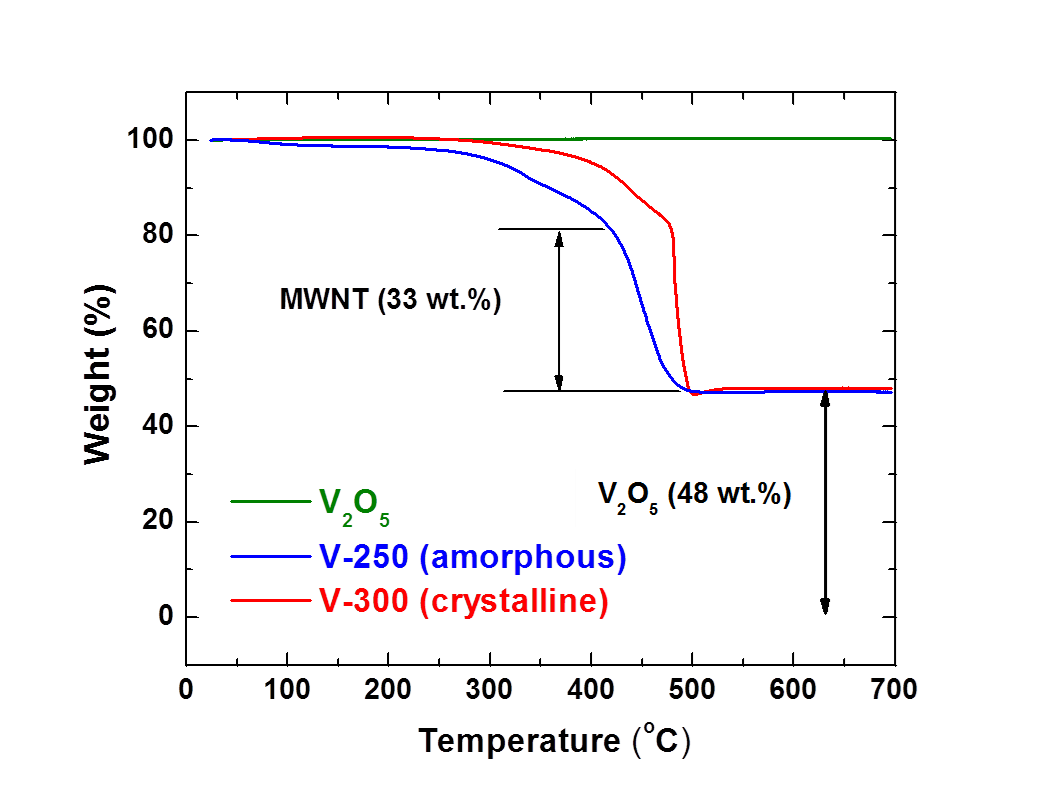


**Figure S2.** TGA profiles of V-250 and V-300 electrode sheets. From these TGA results and the selective etching of PAN (dimethylformamide (DMF) was used as an etching agent), the composition ratio of the electrode sheets was estimated to be (V_2_O_5_/MWNT)/PAN = (48/33)/19 (w/w/w).


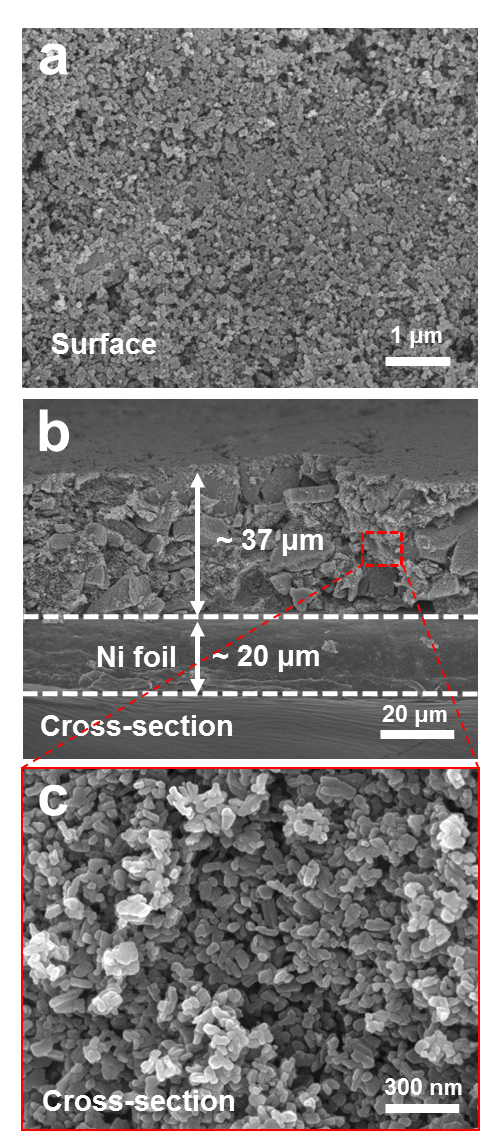


**Figure S3.** SEM images of control V_2_O_5_ electrode sheet (V_2_O_5_ nanoparticles/carbon black additive/PVdF binder = 70/20/10 (w/w/w) on a Ni foil current collector, fabricated by conventional slurry casting method): (a) Surface. (b) Cross-section. (c) Cross-section (high-magnification).


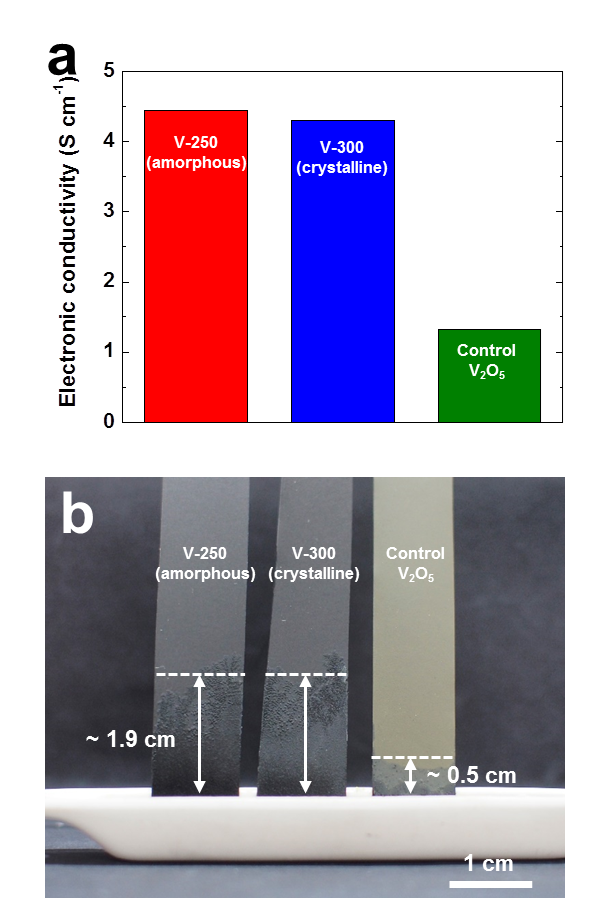


**Figure S4.** Comparison in the physical properties between the V-250 and control V_2_O_5_ electrode sheet: (a) Electronic conductivity. (b) Electrolyte (= 2 M KCl aqueous electrolyte) wettability (expressed as electrolyte-immersion height).


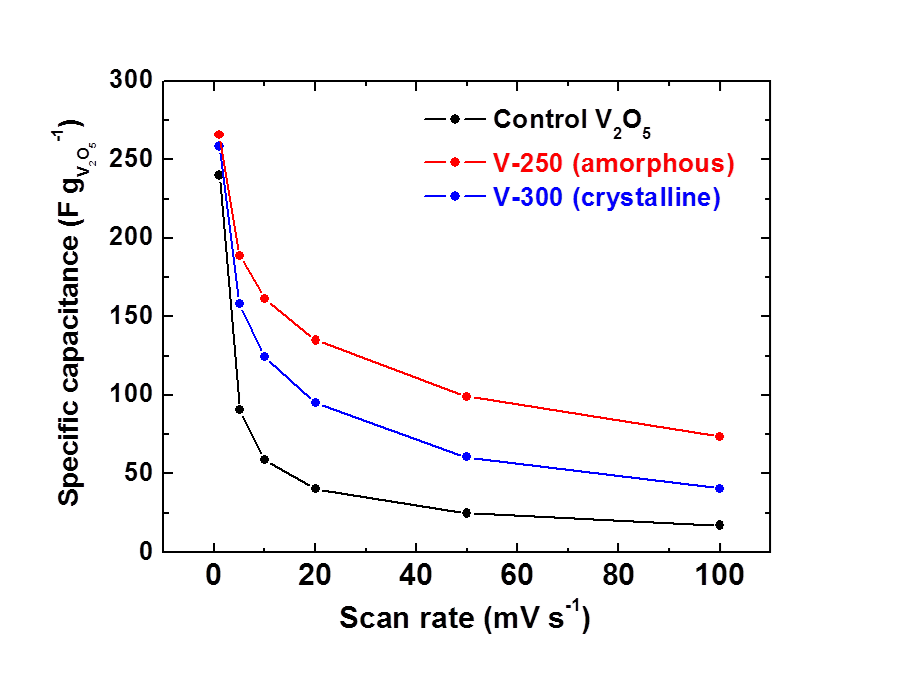


**Figure S5.** Comparison in the (V_2_O_5_ powder weight-based) specific gravimetric capacitance (F g_V2O5_^-1^) between the electrode sheets as a function of scan rate (1 – 100 mV s^−1^).


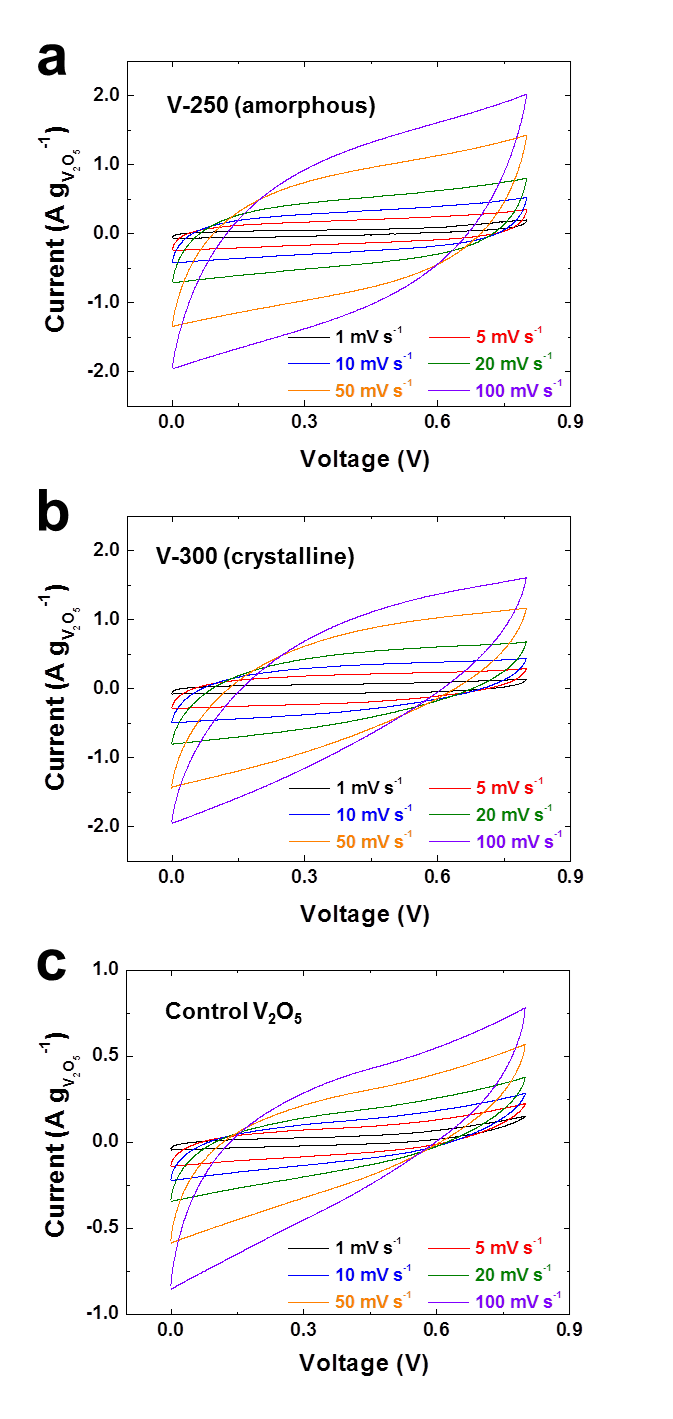


**Figure S6.** Cyclic voltammetry (CV) curves of the electrode sheets over a wide range of scan rates (1 – 100 mV s^−1^) of: (a) V-250; (b) V-300; (c) control V_2_O_5_.


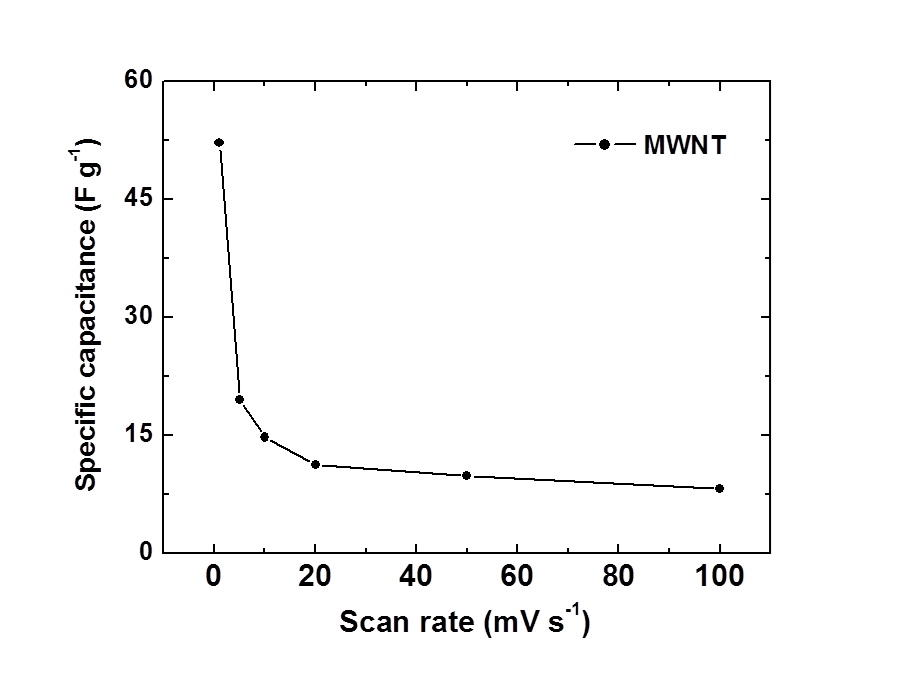


**Figure S7**. Specific gravimetric capacitance of the MWNT electrode sheet (MWNTs/PAN nanofibers = 61/29 (w/w), without V_2_O_5_ powders) over a wide range of scan rates (1 – 100 mV s^−1^).


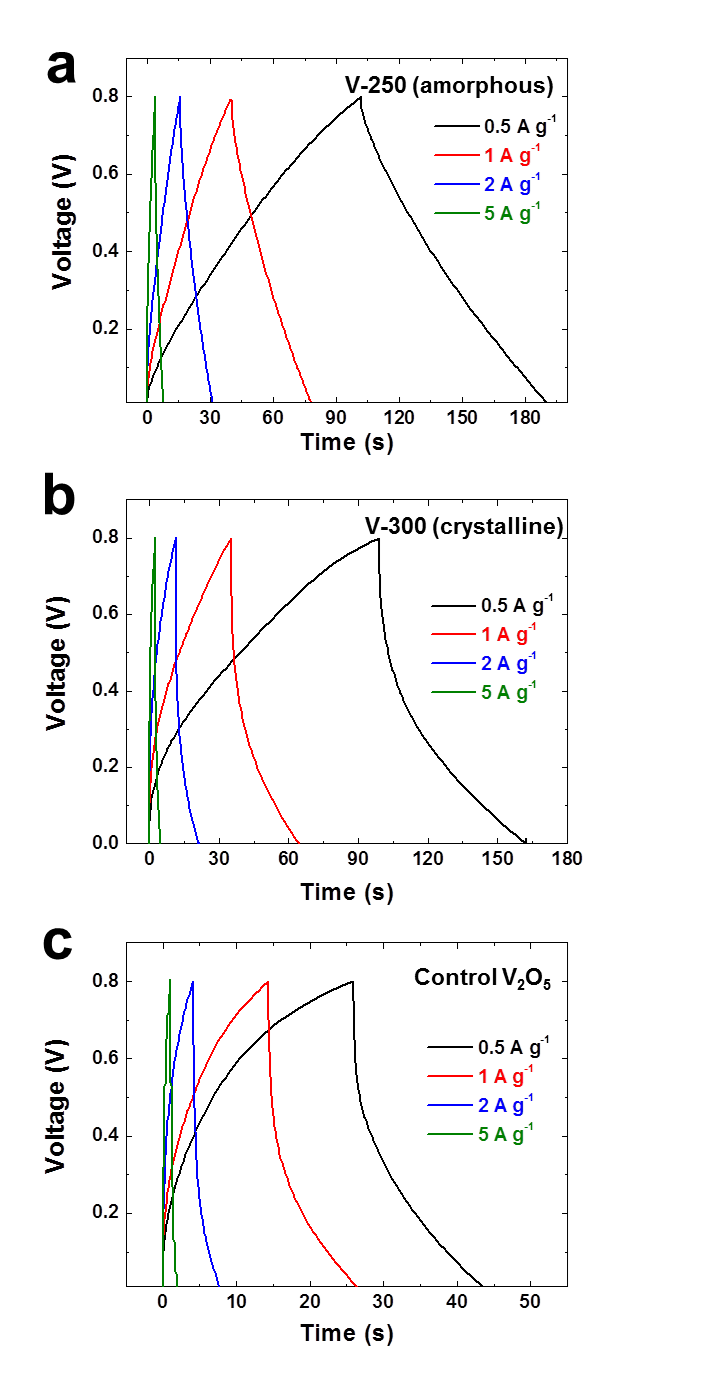


**Figure S8.** Galvanostatic charge/discharge (GCD) profiles of the electrode sheets over a wide range of current densities (0.5 – 5.0 A g^−1^) of: (a) V-250; (b) V-300; (c) control V_2_O_5_.


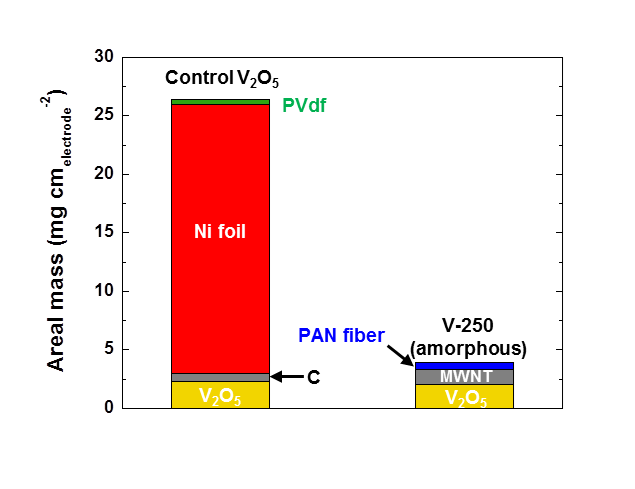


**Figure S9.** Comparison in the areal mass (mg cm_electrode_^-2^) between the V-250 and control V_2_O_5_ electrode sheets in terms of their components.


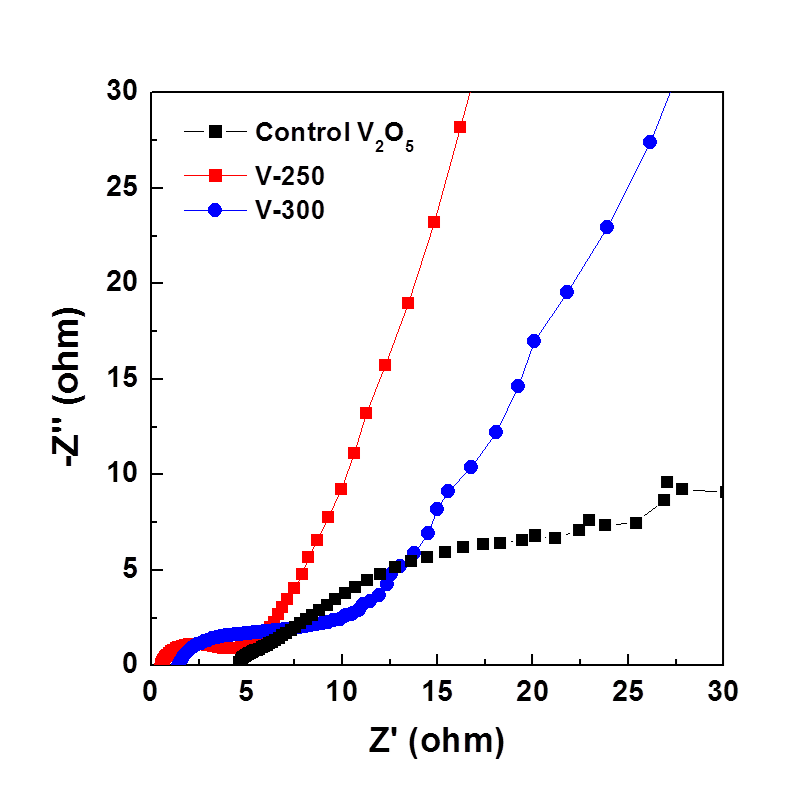


**Figure S10**. AC impedance spectra of the V-250, V-300, and control V_2_O_5_ sheets.

**Table S1.** Comparison in the specific capacitance between this work and the previously reported V_2_O_5_/CNT composites.

| **Electrode**  **(Current collector)** | **V_2_O_5_ materials-based specific capacitance** | **Cell configuration** | **Reference** |
| --- | --- | --- | --- |
| V-250 electrode sheet  (Current collector-free) | 302 F g^-1^ at 1 mV s^-1^ | Symmetric | This work |
| V_2_O_5_/f-MWCNT hybrid  (Ni foam) | 467 F g^-1^ at 2 mV s^-1^  85 F g^-1^ at 2 mV s^-1^ | Three-electrode  Symmetric | *RSC Adv.* **4,** 37437–37445 (2014) |
| V_2_O_5_/CNT composites  (ITO glass) | 116 F g^-1^ at 0.1 A g^-1^ | Symmetric | *ChemElectroChem* **3,** 158–164 (2016) |
| MWCNTs/V_2_O_5_  (Graphite sheet) | 535 F g^-1^ at 5 mV s^-1^ | Three-electrode | *Sci. Rep.* **5,** 15551 (2015) |
| V_2_O_5_-coated MWCNTs  (Glassy carbon) | 510 F g^-1^ at 1 mV s^-1^ | Three-electrode | *Electrochim. Acta* **111,** 400–404 (2013) |
